# *NOD2* mosaicism in Blau syndrome

**DOI:** 10.1186/1546-0096-13-S1-P59

**Published:** 2015-09-28

**Authors:** A Mensa-Vilaro, J De Inocencio, W Tarng Cham, E Gonzalez-Roca, P Tejada-Palacios, S Ping Tang, E Ruiz-Ortiz, E Enriquez-Merayo, S Chin Lim, G Magri, S Plaza, MC Anton, A Cerutti, R Ariffin, J Yagüe, JI Arostegui

**Affiliations:** 1 Hospital Clínic, Immunology, Barcelona, Spain; 2 Hospital 12 de Octubre, Pediatric Rheumatology, Madrid, Spain; 3 Selayang Hospital, Kuala Lumpur, Malaysia; 4 Institut Municipal d'Investigació Mèdica, Barcelona, Spain; 5 Kuala Lumpur Hospital, Kuala Lumpur, Malaysia

## Introduction

Gene mosaicism describes an individual who has developed from a single zygote and has two or more cell types with distinct genotypes. Three major types of mosaicism have been described and are referred as gonadal, somatic and gonosomic mosaicism. They mainly differ on the body distribution of the somatic mutation and in their clinical consequences. The recent use of next-generation sequencing technologies is revealing the relevant role of gene mosaicism in various diseases other than cancer, including monogenic autoinflammatory diseases such as cryopyrinopathies and STING-associated vasculopathy with onset in infancy [1-3].

## Objectives

To describe the first known cases of somatic and gonosomic *NOD2* mosaicism in Blau syndrome (BS).

## Patients and methods

Genomic DNA was extracted from hematopoietic and non-hematopoietic cell types. *NOD2* analyses were performed by both Sanger sequencing and targeted deep sequencing (TDS).

## Results

The first patient is a 17-year-old Moroccan girl who presented with bilateral red eye at the age of 3 years. Severe panuveitis, erythematous rash and oligoarthritis were observed during the following years. Sanger sequencing did not clearly detect any disease-causing *NOD2* mutation. However, a careful analysis of Sanger chromatograms revealed a potential c.1001G>A transition that might cause the appearance of the well-known p.Arg334Gln *NOD2* mutation. TDS confirmed the presence of somatic *NOD2* mosaicism by means of the detection of the somatic p.Arg334Gln mutation in hematopoietic and non-hematopoietic tissues with variable frequencies (4.9-11.0%).

The second patient is a 37-year-old Malaysian male suffering from skin rash and bilateral uveitis since the age of 22 years. He has two affected daughters, aged 4- and 2-year-old, with the classical BS triad (skin rash, polyarthritis and uveitis). Sanger *NOD2* sequencing revealed the germline p.Arg334Gln mutation in both daughters, and unexpectedly its absence in the father. Further analysis of father's Sanger chromatograms suggested the presence of the p.Arg334Gln mutation as a potential somatic mutation. TDS confirmed the presence of gonosomic *NOD2* mosaicism in the father by means of the detection of the somatic p.Arg334Gln mutation in different tissues with different allele frequencies (0.9-12.9%).

## Conclusions

We describe for the first time the involvement of *NOD2* mosaicism in BS pathogenesis by using the novel TDS technology. Our findings clearly support that gene mosaicism might have a relevant role in either “mutation-negative” BS patients or in parents of an affected child with an apparent *de novo* germline mutation.

## Consent to publish

Written informed consent for publication of their clinical details was obtained from the patient/parent/guardian/relative of the patient.
